# Detection of Rift Valley Fever virus inter-epidemic activity in Kilimanjaro Region, North Eastern Tanzania

**DOI:** 10.1080/16549716.2021.1957554

**Published:** 2021-08-20

**Authors:** Medard S Kumalija, Jaffu O Chilongola, Rule M. Budodo, Pius G. Horumpende, Sixbert I. Mkumbaye, John-Mary Vianney, Richard S. Mwakapuja, Blandina T. Mmbaga

**Affiliations:** a Department of Global Health and Biomedical Sciences, Nelson Mandela Institution of Science and Technology, Arusha, Tanzania; b Department of Medical Biochemistry and Molecular Biology, Kilimanjaro Christian Medical University College, MoshiTanzania; c Department of Clinical Trials, Kilimanjaro Clinical Research Institute, Moshi, Tanzania; d Department of Public Health and Research, Lugalo Military College of Medical Sciences, Dar Es Salaam, Tanzania; e Department of Bacterial Vaccines, Tanzania Veterinary Laboratory Agency (TVLA), Kibaha, Tanzania

**Keywords:** Epidemiology, seroprevalence, inter-epidemic, Rift Valley Fever, Tanzania

## Abstract

**Background:**

Rift Valley Fever virus (RVFV) is a zoonotic arbovirus of public health impact infecting livestock, wildlife, and humans mainly in Africa and other parts of the world. Despite its public health importance, mechanisms of RVFV maintenance during interepidemic periods (IEPS) remain unclear.

**Objective:**

We aimed to examine comparatively exposure to RVFV between humans and goats and RVFV infection between humans, goats and mosquitoes.

**Methods:**

A cross sectional study was performed in the Lower Moshi area of the Kilimanjaro region from March to June 2020. RVFV exposure was determined by detecting IgG/IgM to RVFV using a competitive enzyme linked immunosorbent assay whereas infection was determined by real time quantitative polymerase chain reaction (RT-qPCR) assay.

**Results:**

Results show that the male gender was related to RVFV seropositivity (χ^2^ = 5.351; p=0.030). Being 50 years and above was related to seropositivity (χ^2^ =14.430; p=0.006) whereas bed net use, larger numbers of persons living in the same house (>7 persons) and RVFV seropositivity in goats were related to higher seropositivity to RVFV among humans χ^2^ =6.003; p=0.021, χ^2^ =23.213; p < 0.001 and χ^2^ =27.053; p < 0.001), respectively. By the use of RT-qPCR, goats exhibited the highest RVFV infection rate of 4.1%, followed by humans (2.6%), *Ae. aegypti* (2.3%), and *Cx. pipiens* complex(1.5%). Likewise, a higher proportion of goats (23.3%) were RVFV seropositive as compared with humans (13.2%).

**Conclusion:**

Our findings suggest the Lower Moshi area as a potential hotspot for Rift Valley Fever (RVF), posing the danger of being a source of RVFV spread to other areas. Goats had the highest infection rate, suggesting goats as important hosts for virus maintenance during IEPs. We recommend the implementation of strategies that will warrant active RVF surveillance through the identification of RVF hotspots for targeted control of the disease.

## Background

Rift Valley Fever virus (RVFV) is a zoonotic arbovirus affecting livestock and humans mainly in Africa and the Arabian Peninsula [1–4], although recent reports indicate the presence of Rift Valley Fever (RVF) in other parts of the world [5]. According to the World Health Organization (WHO), RVF is a priority disease due to its considerable public health impact in areas where it occurs and the inadequate interventions to control it [6]. It is also considered an important threat to agriculture in African countries, including Tanzania [7–9]. Transmission of RVFV to animals is mainly through bites by infected *Aedes* and *Culex* mosquitoes, whereas human transmission is largely accomplished through direct contact with tissues of RVFV-infected animals [10]. Most people infected by RVFV remain asymptomatic, although a small percentage present with clinical disease [11–13].

Previous studies had suggested that maintenance of the virus in its host animals during inter-epidemic periods (IEPs) is driven by recurrent introduction of the virus from ‘hotspot’areas to areas with less favorable conditions through animal movements [14]. Disease pathogenesis, pathology and endemic maintenance within mammalian hosts have also been described [15,16]. Some explanations have been made regarding the possible mechanisms by which the virus is maintained during IEPs. Previous work has also pointed out low levels of RVFV exposure as a key mechanism of virus maintenance [17,18]. Other reports have hypothesized critical mechanisms for survival of RVFV during long inter-epizootic periods as vertical transmission through mosquito eggs to mosquito offspring [19–21].

Further, some literature asserts the maintenance of RVFV to depend on the presence of competent vectors, hosts and other factors such as sufficient livestock density, rainfall providing vector breeding sites, and temperatures that support vector development and pathogen replication [22]. All of these hypothesesare graphically summarized in Figure 3. However, there is apparent paucity of information about the role of animals, humans and vector mosquitoes in maintaining the virus during IEPs. The maintenance mechanisms during IEPs become interesting due to the absence of a clear understanding of where the virus hides during the ‘silent’ periods. Differential exposure of RVFV in high-risk agropastoral communities in North Eastern Tanzania has not been examined. We aimed to determine comparatively the prevalence of antibodies to RVFV in humans and goats, and RVFV infection among humans, goats and mosquitoes in an agropastoral community in the Lower Moshi area of Moshi Rural district.

## Methods

### Study design and site

**Figure 1.**
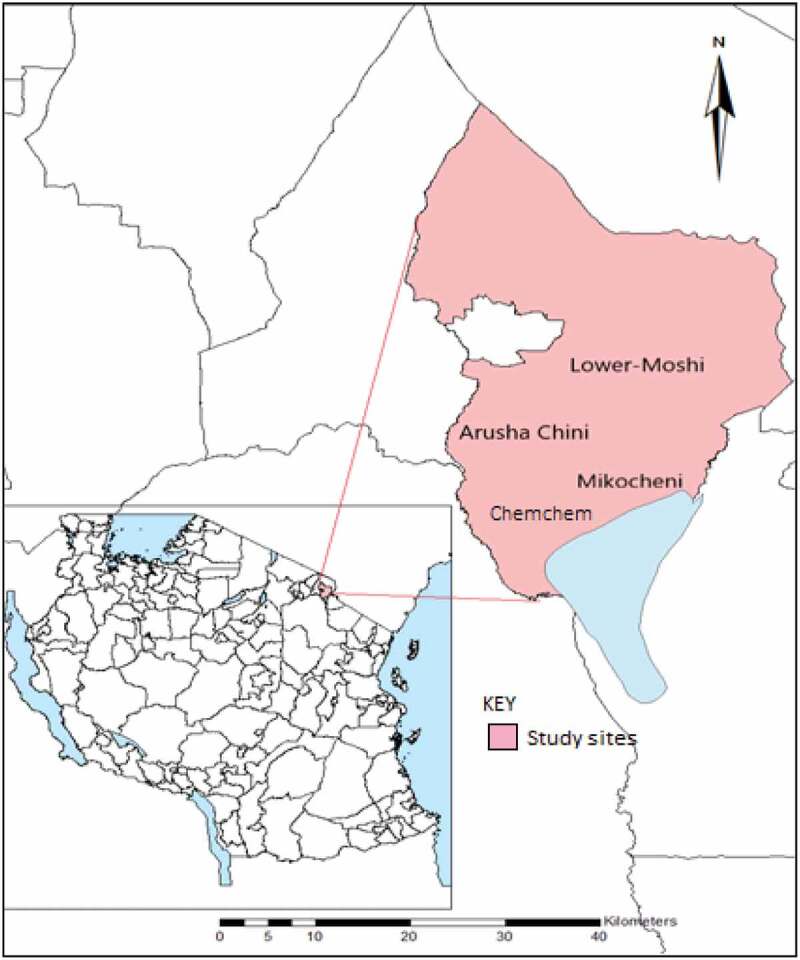
Map of Tanzania showing the study site

A community-based, cross-sectional survey was conducted in three villages of Lower Moshi in Moshi Rural district, Kilimanjaro region of Tanzania. Data were collected between March and June 2020 involving three villages, namely Mikocheni, Chemchem, and Arusha Chini. This period of the year was chosen for sampling because it experiences the long rains that are associated with increased mosquito activity in the area. Lower Moshi is located on the southern foothills of Mount Kilimanjaro (Figure 1). On the west, Lower Moshi is bordered by the Kikuletwa River, Hai District, and Manyara Region. To the east Lower Moshi borders Mwanga district. Lower Moshi elevation ranges between 700 and 800 m above sea level. The main RVF vectors in this area are *Culex spp* and *Aedes spp* [23]. Numerous water streams cross the area and they form the irrigation channels for rice and sugar cane. The rice irrigation schemes have structured and unstructured canal networks, covering an area of about 1100 hectares. During the rainy season, temporary pools that serve as RVFV vector breeding sites are formed. Their persistence beyond the rains contributes to further RVFV transmission [24]. The area has two rainy seasons, the long rains which run from March to May and the short rainy season from November to December. The average annual rainfall is about 900 mm per year [25].

## Participants and sample collection

### Human participants

Participants in this study were males and females aged between 10 and 70 years from 266 households. Participants aged ≥18 years were either smallholder crop farmers or livestock keepers. Consent to participate in the study was obtained from adults aged ≥18 years whereas parents or legal guardians for participants aged <18 years assented on their children’s behalf.

### Animals

Animal sampling was carried out by animal health experts from the Tanzania Veterinary Laboratory Agency (TVLA). Up to 15 goats were selected from each herd using a systematic sampling technique whereby every third and fifth animal was included depending on the size of the herd. In total, 2986 goats from 120 herds from the 266 households were sampled in the study, an average of 11 goats per household.

### Collection of blood sample from humans and goats

Human blood sampling was done by expert phlebotomists from the Kilimanjaro Christian Medical Center (KCMC). Three milliliters of human blood were collected from the median cubital vein by venipuncture. Selected animals were manually restrained and 3 ml of blood were collected through the jugular venipuncture using a sterile vacutainer needle. Each sample from both animals and humans was divided into two aliquots of 1.5 ml each, and aliquots placed into plain and EDTA vacutainer tubes, respectively. To each sample in EDTA tubes, 4.5 ml of Tri Reagent (Zymo Research, Irvine, CA, USA) were added. The mixture was gently mixed by shaking for 1 min and immediately shipped to the KCRI biotechnology laboratory at 4°C for RNA extraction and polymerase chain reaction (PCR) analyses. Samples in plain tubes were allowed to clot for a maximum of 20 min at room temperature before they were spinned at 2000 × *g* for 10 min in a refrigerated centrifuge to obtain clear serum that was transferred to clean sterile serum tubes. Serum samples from both humans and goats were tested for the presence of IgG/IgM to RVFV. Blood samples from humans and goats that were positive by serology were subjected to PCR analysis. Demographic data from participants were collected using electronic forms designed using Open Data Kit (ODK) tools (https://opendatakit.org/) deployed in Android tablets.

### Mosquito trapping

A BG Sentinel trap (BGS) (Biogents AG, Regensburg, Germany) was used to target outdoor host-seeking adult mosquitoes, particularly *Aedes spp*, *Ochlerotatus spp*, *Culex spp*, *Mansonia spp*, and *Anopheles spp* [26]. BGS traps were used in combination with the BGS-Lure, a dispenser that releases emanations such as those found on human skin (lactic acid, ammonia, and caproic acid) [27]. The BGS-Trap, developed by BioGents GmbH (Regensburg, Germany), consists of an easy-to-transport, collapsible white bucket with white gauze covering its opening. In the middle of the gauze cover there is a black tube through which a downflow is created by a 12 V DC fan that causes any mosquito in the vicinity of the opening tube to be sucked into a catch bag [27]. Mosquitoes were immediately morphologically identified in the field and consequently sorted according to their genera. Culex mosquitoes were grouped together and denoted as *Cx. pipiens* complex. Owing to the small number of other mosquito genera trapped, two key genera, *Cx. pipiens* complex and *Ae. aegypti*, were subjected to RT-qPCR analyses for RVFV RNA in pools of 50 s. For *Ae. aegypti* and *Cx. pipiens* complex, 347 and 130 mosquito pools were collected and analyzed, respectively.

## Laboratory procedures

### RVFV competitive ELISA

All serum samples were tested for the presence of antibodies against RVFV using a competitive ELISA (cELISA) using the ID Screen RVF Competition Multi-Species kit (ID-vet, Grables, France), which detects both IgG and IgM antibodies directed against the RVFV nucleoprotein (NP). Validation tests for the test kit have shown a sensitivity of between 91% and 100% and a specificity of 100% [28]. The cELISA was performed according to the instructions of the manufacturer and as described previously [29,30]. To control the validity of each plate, the mean value of the two negative controls (OD_NC_) was computed whereby a plate was considered valid if the OD_NC_ was >0.7. For a valid plate, the mean value of the two positive controls divided by OD_NC_ had to be <0.3. For each sample, the competition percentage was calculated by dividing (OD_sample_/OD_NC_) × 100. If the value was ≤0.4 the sample was considered to be positive, while a value >0.5 was considered negative. Only samples that tested positive for cELISA were subjected to RT-qPCR for RVFV detection.

### RNA detection by PCR, purification, and real-time PCR amplification

For detection of RVFV RNA in humans and goats, RNA was extracted from Trizol archived blood in EDTA tubes using a DirectZol miniprep kit (Zymo Research, Irvine, CA, USA) by using the Boom method [31]. To isolate RVFV RNA from mosquitoes, pools of 50 unfed monospecific female mosquitoes were placed in cryovials and transferred into Lysing Matrix impact-resistant tubes containing 1.4 mm ceramic beads (MP Biomedicals, CA, USA). Samples were disrupted by bead beating at 10,000 × *g* for 1 min and spun at 1000 × *g* for 10 min at 4°C. The supernatant was transferred into labeled RNase-free tubes. Purification procedures were done using a Direct-zol™ RNA miniprep kit (Irvine, CA, USA) following the manufacturer’s instructions.

For both human/goat and mosquito samples, RNA concentration and quality check were performed using a NanoDrop™ 2000 Spectrophotometer (Thermo Scientific, NY, USA) before storage at −80°C. RVFV RNA was detected using TaqMan probe-based one-step RT-qPCR targeting the RVFV Gn gene as described by Gudo and colleagues [2] using the Applied Biosystems ViiA7 PCR platform (Thermo Scientific, NY, USA).

### Nature of data and data analysis

Data analysis was performed using IBM SPSS v.26 (IBM® Corp., Armonk, NY, USA). Descriptive data were presented as frequencies and percentages, means, and medians. Categorical data were reported as a tabulation of proportions and compared between humans and goats. The chi-squared statistic (χ^2^) was used to examine associations between seropositivity to RVFV and RVFV infection in both humans and goats. Mean IgM and IgG concentrations were compared between humans and goats by paired t-test. Percent positivities to RVFV infection in goats, humans, and mosquitoes were reported as histograms.

### Ethical issues

This study obtained approval from the Kilimanjaro Christian Medical University College (KCMUCo) Research and Ethics Committee (CRERC) with approval certificate #2419. Permission to conduct this study was also obtained from the Kilimanjaro Regional and District Administrative Secretaries, District Medical and Veterinary Officers, and local village and ward executive officers of respective villages. Before commencement of sample collection, written informed consent was obtained from all study participants aged 18 years and above by signing ‘informed consent’ forms, whereas parents and/or legal guardians of participants under 18 years and participants who could not read or write signed the ‘informed consent’ on their behalf. All authors hereby confirm that all procedures in this study were approved by CRERC and were performed in accordance with the ethical standards as laid down in the 1964 Declaration of Helsinki. The authors also confirm that all procedures that involved animals in this study were conducted in compliance with the ARRIVE guidelines.

## Results

### Demographic characteristics of human participants

A total of 266 human participants were enrolled in the study. Of the participants enrolled, more than half (56.4%) were females. The median age (interquartile range) of participants was 45 (30–55). The majority of participants (74.4%) came from households with more than four people in the same house. With regards to the participants’ education, 63.2% of participants had attained primary school education. Most participants (72.9%) kept livestock (cattle, sheep, goats, and/or chickens). Nearly one-quarter (24.8%) of the participants reported they had used an insecticide-treated bed-net (ITN) the night before the interview (Table 1).

**Table 1.** Demographic characteristics of human participants

Characteristics	n	%
Age group (years)		
≤20	28	10.5
21–50	140	52.7
>50	98	36.8
(Median, IQR) years	45 (30–55)	
Sex		
Male	116	43.6
Female	150	56.4
Individuals living in a household		
<4	68	25.6
≥4	198	74.4
Highest education		
No formal education	51	19.2
School education (primary/secondary)	168	63.2
Tertiary education (college)	47	17.7
Type of animals kept by the participant		
None	72	27.1
Chicken only	82	30.8
Goats and sheep only	54	20.3
Cattle only	55	20.7
Goats, sheep, cattle, and chicken	3	1.1
ITN use*		
Yes	66	24.8
No	200	75.2

IQR, Interquartile range; *72 missing entries.

### Demographic factors of goats involved in the study

A total of 2986 goats were sampled, of which 1590 (53.25%) were aged less than 12 months and about one-third (30.1%) were males. Of the 120 sampled herds, 74 (61.7%) comprised 20 goats or fewer (Table 2).

**Table 2.** Age, sex and number of herds of goat sampled (n = 2986)

Characteristics	n	%
Age category (months)		
<12	1590	53.25
>12	1396	46.75
Sex		
Male	899	30.1
Female	2087	69.9
Herd size*		
<20	74	61.7
20–50	24	20.0
>50	22	18.3

*Total sampled herds = 120.

### RVF seroprevalence in humans and goats

Results for human seropositivity to RVF are summarized in Table 3. Out of the 34 RVF seropositive persons, 19 (55.9%) were males. Across age groups, 71.4% of individuals aged 50 years and above were seropositive for RVF. When seropositivity was analyzed across human RVFV PCR positivity, 28 of the 35 (80%) samples tested for RFVF by PCR were seropositive for RVF antibodies whereas 22 (62.9%) of seropositive individuals had not used an ITN in the last 48 h. Seventeen of the 35 (48.6%) RVF seropositive individuals were from households with a larger number (above seven) of individuals living in the same house. Twenty-five of 35 RVFV seropositive individuals (71.4%) did not travel outside the study area. Six of the 10 RVF seropositive individuals (60.0%) who had traveled outside the study area had traveled to a rural destination. The majority (71.4%) of the seropositive individuals had a school (primary/secondary) education. For goats tested for RVFV by PCR, eight out of the nine goats (89.9%) that were seropositive for RVF were PCR negative.

**Table 3.** Factors associated with RVFV seropositivity in humans

Variable	Level	Negative, n (%)	Positive, n (%)	χ^2^(p)
Sex	Male	47 (34.3)	19 (55.9)	
	Female	90 (65.7)	15 (44.1)	5.351 (0.030)
Age group	11–20	18 (13.1)	1 (2.9)	
	21–30	26 (19.0)	2 (5.7)	
	31–40	23 (16.8)	3 (8.6)	
	41–50	19 (13.9)	4 (11.4)	
	>50	51 (37.2)	25 (71.4)	14.430 (0.006)
Human RVFV PCR positivity	Positive	0 (0.0)	7 (20.0)	
	Negative	1 (100.0)	28 (80.0)	0.248 (1.000)
ITN use	Yes	24 (17.9)	13 (37.1)	
	No	110 (82.1)	22 (62.9)	6.003 (0.021)
Number of persons in a household	1–3	45 (33.1)	5 (14.3)	
	4–6	74 (54.4)	13 (37.1)	
	7+	17 (12.5)	17 (48.6)	23.213 (<0.001)
Travel outside site	Yes	48 (35.0)	10 (28.6)	
	No	89 (65.0)	25 (71.4)	0.521 (0.551)
Destination of travel	Urban	22 (45.8)	3 (30.0)	
	Peri-urban	10 (20.8)	1 (10.0)	
	Rural	16 (33.3)	6 (60.0)	2.545 (0.346)
Highest education	No formal education	34 (24.8)	8 (22.9)	
	School education (primary/secondary)	91 (66.4)	25 (71.4)	
	Tertiary education (college)	12 (8.8)	2 (5.7)	0.465 (0.830)
RVFV PCR positivity in goats	Yes	2 (66.7)	8 (88.9)	
	No	1 (33.3)	1 (11.1)	0.800 (1.000)
RVFV seropositivity in goats	Yes	106 (77.4)	11 (31.4)	
	No	10,631 (22.6)	24 (68.6)	27.053 (<0.001)
Culex RVFV PCR positivity	Yes	1 (0.7)	33 (94.3)	
	No	136 (99.3)	2 (5.7)	4.041 (0.106)
Aedes RVFV PCR positivity	Yes	0 (0.0)	2 (5.7)	
	No	137 (100.0)	33 (94.3)	7.921 (0.04)
Type of animals kept	None	45 (80.40)	11 (19.60)	
	Chicken	37 (78.70)	10 (21.30)	
	Goats and sheep	29 (76.30)	9 (23.70)	
	Cattle	25 (83.30)	5 (16.70)	
	Goats, sheep, cattle	1 (100.00)	0 (0.00)	0.809 (0.929)

### Factors associated with RVFV seropositivity in humans and goats

Human RVFV seropositivity was analyzed for any associations with participant age, ITN use within the last 24 h, positivity for RVFV infection, number of persons living under the same roof, recent travel outside the study site, highest education of the participant, and RVFV infection and seropositivity in goats. The results are presented in Table 3. The results show that male gender was significantly more related to RVFV seropositivity (χ^2^ = 5.351; p = 0.030). Likewise, participants aged 50 years and above were more seropositive as compared with their younger counterparts (χ^2^ = 14.430; p = 0.006). ITN use, larger numbers of people living in the same house (>7), and RVFV seropositivity in goats were related to higher seropositivity to RVFV (χ^2^ = 6.003; p = 0.021, χ^2^ = 23.213; p < 0.001 and χ^2^ = 27.053; p < 0.001), respectively. Among the selected factors analyzed for possible association with IgM/IgG RVFV seropositivity in goats, only IgM/IgG RVFV seropositivity in humans had a significant relationship (χ^2^ = 27.053; p < 0.001). *Ae. aegypti* infection by RVFV (RVFV PCR positivity) was strongly associated with human RVF seropositivity (χ^2^ = 7.921; p = 0.04) (Table 4).

**Table 4.** Factors associated with RVFV seropositivity in goats

Variable	Level	Negative, n (%)	Positive, n (%)	χ2 (p)
RVFV PCR positivity in goats	Positive	3 (75.0)	8 (89.9)	
	Negative	1 (25.0)	1 (11.1)	0.410 (1.000)
Herd size	<20	19 (39.6)	12 (41.4)	
	20–50	16 (33.3)	8 (27.6)	
	>50	13 (27.1)	9 (31.0)	0.305 (0.874)
IgM/IgG seropositivity in humans	Positive	11 (9.4)	24 (43.6)	
	Negative	106 (90.6)	31 (56.4)	27.053 (<0.001)
RVFV PCR positivity in humans	Positive	2 (16.7)	5 (20.8)	
	Negative	10 (83.3)	19 (79.2)	0.089 (1.000)
Sex	Male	17 (34.0)	12 (41.4)	
	Female	33 (66.0)	17 (58.6)	χ^2^ = 0.430 (0.629)
Age	0–12 months	28 (56.0)	15 (51.7)	
	>12 months	22(44.0)	14 (48.3)	χ^2^ = 0.135 (0.216)

### RVFV PCR positivity in human, goat and mosquitoes and RVFV seropositivity in humans and goats

**Figure 2.**
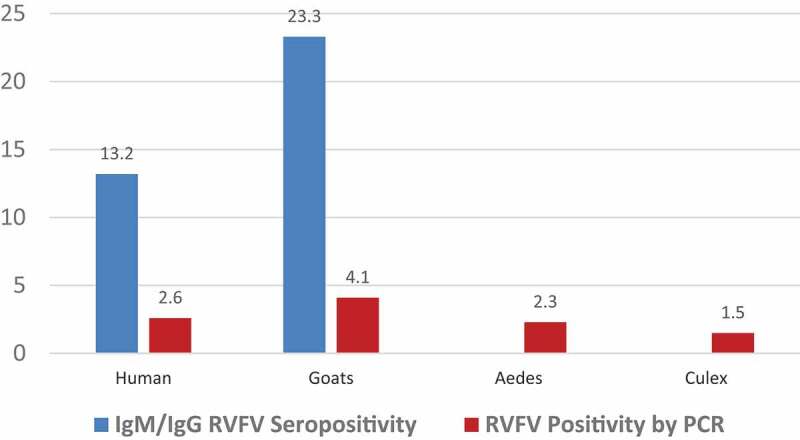
RVFV PCR positivity in humans, goats and mosquitoes and RVFV seropositivity in humans and goats

Percentages of RVFV seropositive humans and goats as well as PCR results for viral infections were determined (Figure 2). Compared with humans, goats were more seropositive to RVFV (23.3% seropositive goats against 13.2% seropositive humans). *Ae. aegypti and Cx. pipiens* complex were the dominant species among collected mosquitoes. However, *Mansonia spp* and *Anopheles spp* mosquitoes were also collected in smaller numbers. Our analyses were focused on *Ae. aegypti* and*Cx. pipiens* as the main documented vectors for RVFV. When virus detection was performed using RT-qPCR, goats exhibited the highest infection rate of 4.1%, followed by humans (2.6%). For *Ae. aegypti*, 347 monospecific pools of 17,350 mosquitoes were tested while for *Cx. pipiens* complex 130 pools of 6500 mosquitoes were tested. Out of these, eight (2.3%) *Ae. aegypti* pools were positive while only two (1.5%) *Cx. pipiens* complex pools were PCR positive for RVFV. The minimum infection rates (MIRs) were computed by using the formula [Number of positive pools/Number of mosquitoes tested] × 1000, giving 0.461 and 0.308 for *Cx. pipiens* complex and *Ae. aegypti*, respectively.

**Figure 3.**
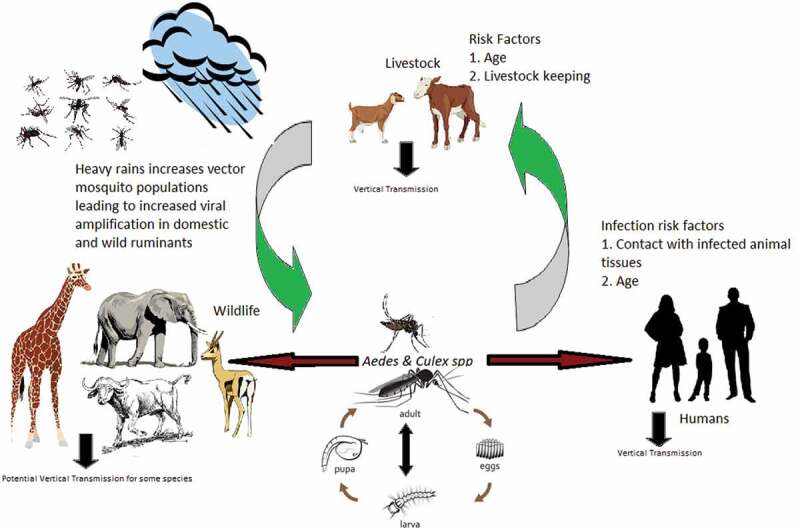
Hypothetical diagram to show the transmission and maintenance mechanisms for RVFV in the study area

## Discussion

The main aim of this study was to examine the degree of exposure to RVFV in goats and humans. This study also sought to detect RVFV in humans, goats, and key RVFV vector mosquitoes, *Ae. aegypti and Cx. pipiens* complex. Results from this study show that, although there has been no RVF outbreak reported in Tanzania since 2006–2007, antibodies to RVFV and the virus have been detected in humans and goats in the Lower Moshi area. Findings from this study indicate that 13.2% and 23.3% of tested humans and goats, respectively, had circulating antibodies to RVFV. Our findings emphasize an active exposure to RVFV during IEPs as previously reported by some studies across geo-ecological zones of Tanzania [23,32–35].

In this study, goats had higher exposure rates to RVFV compared with humans. Although *Ae. aegypti*, the major vector for RVFV, is known to be anthropophilic in nature, it has also been reported to have both exophagic and exophilic feeding behaviors [36,37,]. Consequently, this behavior can be implicated as a key behavior in its role as a vector for many zoonotic infections. Despite its preference for human hosts [36,38,39], we report higher RVF seropositivity in goats. The transmission of RVFV is not absolutely dependent on the presence of vector mosquitoes. Direct human contact with infected animal tissues has been reported as a significant factor for its transmission from animals to humans [23,40,41]. Not all of the human participants in this study were directly involved in activities that increased their direct contact with infected tissues such as infected aborted fetuses, and those working in slaughterhouses, which could partly explain the lower seropositivity to RVFV in humans compared with goats. Furthermore, a much more intense interaction between RVFV vector mosquitoes and goats exists in the studied agropastoral community, exposing goats to higher RVFV exposure and infection rates. Clinically, goats could be the main source of RVFV infection in humans rather than vice versa.

In the current study, RVFV RNA was detected in humans, goats, and mosquitoes. Goats exhibited the highest infection rate of 4.1%, followed by humans (2.6%). Viral RNA was also detected in 2.3% and 1.5% of tested *Ae. aegypti* and *Cx. pipiens* complex mosquito pools. This study was conducted to shed light on the maintenance mechanisms of RVFV by investigating both exposure and infection rates in mammalian and arthropod vectors. To our knowledge, this is the first study conducted in Tanzania to report concomitantly on RVFV diagnosis in humans, animals, and mosquitoes. Many of the previous studies that sought to understand the epidemiology of RVFV in Tanzania either focused on sero-epidemiology or could not detect RVFV RNA in mammalian and arthropod vectors [37].

The detection of RVFV RNA in some of the human, goat and mosquitoes demonstrated evidence of recent and active RVFV infection in the area. This observation further supports the increasing body of evidence pertaining to RVFV transmission during the IEPs in sheep and goats. The detection of IEP RVFV by PCR is suggestive of active RVFV transmission in the study area, in the absence of reported clinical cases. This brings forth the questions as to whether the disease is overlooked due to lack of surveillance systems, or whether there are other RVFV strains with low pathogenicity that do not lead to clinical disease. This is a likely explanation since most RVFV infections are either subclinical or misdiagnosed as other infections [34].

Although the interactions of arboviruses and their vectors are complex and their epidemiology is poorly understood, our findings support the hypothesis that during IEPs RVFV is likely maintained by localized low-level transmission between mosquito vectors and mammalian hosts without any noticeable clinical symptoms [19,23,42,43]. *Cx. pipiens* complex mosquitoes were less infected by RVFV than *Ae. aegypti*, which could mean the role of the latter as a key vector and means of RVFV maintenance in the area. This finding is similar to other studies [44] that have reported *Ae. aegypti* to be more susceptible to RVFV infection compared with other known RVFV vectors. Evidence for RVFV transmission during IEPs has previously been reported among humans, livestock, and wild animals in Tanzania and elsewhere [1–4,6,23,30,32–35,37,42,45–48].

Some factors were significantly associated with seropositivity to RVFV in humans, including male gender, more than four people in a household, being older than 50 years, not using an ITN, and higher RVFV seropositivity in goats. RVFV seropositivity in humans was consequently associated with seropositivity in goats. Males, especially in agropastoral communities, seem to be more active outdoors for various subsistence activities, including farming and grazing, which bring them into frequent contact with RVFV susceptible or infected animals. Although we investigated only *Cx. pipiens* complex and *Ae. aegypti*, the potential of other mosquito species such as *Anopheles spp* [49,50] in transmitting RVFV cannot be ignored. Therefore, our findings lay emphasis on the need for continued usage of ITNs, especially among rural and agro-pastoral communities who are more prone to zoonotic diseases.

The study site is characterized by features that are supportive of vector mosquito breeding and intimate human–animal interaction. In the absence of reports on RVFV infection in areas near the study area [23], the detection of antibodies to RVFV in humans and goats and detection of RVFV in humans, goats, and mosquitoes in the study area suggest the site is a potential RVF hotspot [37]. The dominant pastoral grazing system in the study area and surrounding areas is manifested as unlimited movements of livestock as a result of environmental degradation of the wetland due to overstocking and overgrazing increasing the chances of introducing the disease into new areas. The absence of clinical manifestations among livestock and humans in the study area, which could be a consequence of herd immunity or infection by less pathogenic strains, seems to have escaped the knowledge of the veterinary and public health authorities, raising concerns about the available local and national capacity for preparedness and response machinery against zoonotic infections with the potential to cause fatal epidemics. Thus, there exists a critical need for improved surveillance of RVF transmission through detection of RVFV infections in humans, livestock, and vector mosquitoes.

Since passive surveillance of RVF is challenging in the absence of clinical features among humans and livestock, active surveillance is recommended and, where resources may be limited, targeted surveillance in high-risk areas (hotspots) will help prevent future RVF outbreaks. It is critically important to look again at the national contingency plans used in RVF surveillance and response to RVF outbreaks, bearing in mind that observed active transmission of the virus occurs in the absence of expected clinical manifestations that have been the traditional RVF pointers for a long time, such as massive abortions in livestock.

## Conclusion

Here, we have presented data that reveal the presence of anti-RVFV antibodies in humans and goats and the presence of RVFV in humans, goats, and mosquitoes in the Lower Moshi area of the Kilimanjaro region, Northern Tanzania. Collected during a dry season of IEP, our data show that goats had the highest prevalence of antibodies to RVFV and infection rate by the virus, suggesting their key role as reservoirs of RVFV in IEPs. Our findings also point to the Lower Moshi area as being a potential hotspot for RVF, posing the danger of being a source of RVFV to other areas. Consequently, strategies for effective active surveillance of RVF that involve the identification of RVF hotspots for targeted control are recommended. Notwithstanding the strength of our study findings, we acknowledge that our study was limited by a failure to discriminate between IgG and IgM antibodies to RVFV, which was a result of the commercial kit used. Future study designs could circumvent the limitation by testing the antibodies using separate assays.

## Paper context

Recently, no study has identified RVFV in both vertebrates and arthropod vectors in the same setting at the same time. We have shown that goats had the highest prevalence of antibodies to RVFV and infection by the virus, suggesting their key role as reservoirs of RVFV in IEPs. The key message we get from this observation is that the complex transmission of the viruses warrants studies that involve vectors, vertebrate hosts and the viruses.
